# Screening of a Novel Upregulated lncRNA, A2M-AS1, That Promotes Invasion and Migration and Signifies Poor Prognosis in Breast Cancer

**DOI:** 10.1155/2020/9747826

**Published:** 2020-04-11

**Authors:** Kai Fang, Hu Caixia, Zhang Xiufen, Guo Zijian, Lihua Li

**Affiliations:** ^1^Oncology Institute, Affiliated Hospital of Jiangnan University, Wuxi 214062, China; ^2^Department of Oncological Surgery, Affiliated Hospital of Jiangnan University, Wuxi 214062, China

## Abstract

Understanding of prognostic factors and therapeutic targets for breast cancer is imperative for guidance of patient care. We studied 1203 tumour samples from the Gene Expression Omnibus (GEO) to evaluate potential genes related to breast cancer. R software was used to analyse differentially expressed long noncoding RNAs (lncRNAs) in the RNA microarray expression profiles GSE45827 and GSE65216 and to identify a series of differentially expressed lncRNAs associated with human breast cancer. Of these lncRNAs, A2M-AS1, a lncRNA that has not been previously reported, was significantly upregulated in human breast cancer tissues compared with adjacent nontumour tissues. Importantly, A2M-AS1 upregulation was significantly associated with ER-negative, HER2-positive, and basal-like breast cancer and with poor recurrence-free survival and metastasis-free survival in breast cancer patients. After validating these results in 96 collected human breast cancer tissues and 64 paired adjacent noncancerous tissues, we further investigated the roles of A2M-AS1 in human ER-negative and basal-like breast cancer cells. The results revealed that A2M-AS1 significantly promotes human breast cancer cell proliferation, invasion, and migration. Additionally, bioinformatics analysis of genes coexpressed with A2M-AS1 in the context of human breast cancer combined with qRT-PCR and Western blot assays revealed that A2M-AS1 exerts regulatory effects on downstream factors in the cell adhesion molecule pathway, including CD2 and SELL. These results imply that A2M-AS1 might be a promising candidate prognostic factor and therapeutic target for breast cancer.

## 1. Introduction

The most prevalent cancer diagnosed in females is breast cancer [1]. Although clinical breast cancer treatments can achieve favourable results, recurrence and metastasis still account for at least 90% of the mortality due to breast cancer. Therefore, finding new prognostic markers and therapeutic targets is key for effective treatment of breast cancer.

Long noncoding RNAs (lncRNAs) are a class of noncoding RNAs that are longer than 200 nt and have limited protein-coding potential. These molecules play important roles in normal human development and various physiological processes, and dysfunction of lncRNA is associated with a range of diseases, including cancer. lncRNAs participate in tumorigenesis and metastasis through different mechanisms, including epigenetic modification, alternative splicing, RNA decay, posttranslational modification, tumour suppressor gene silencing, and oncogene activation [2–4]. Previous research has revealed that the lncRNA DLEU1 contributes to progression in colorectal cancer via activation of KPNA3 [5]. Besides, the lncRNA PVT1 promotes angiogenesis in gastric cancer by activating the STAT3/VEGFA axis [6]. The lncRNA DSCAM-AS1 is regulated by ER through binding interactions with hnRNPL and promotes oncogenicity in breast cancer, providing insight into resistance to endocrine therapy [7]. However, breast cancer is a highly heterogeneous type of cancer, the occurrence and progression of which are complex processes involving multifactorial induction mechanisms. We hypothesize that many as-yet-unidentified lncRNAs are differentially expressed in breast cancer tissues and adjacent tissues and are closely related to the occurrence and development of breast cancer.

With the advent of the era of “big data,” a variety of public databases have been established. The Gene Expression Omnibus (GEO), which is affiliated with the National Center for Biotechnology Information (NCBI), contains large amounts of high-throughput expression data and related clinical information and is one of the largest and most comprehensive public gene expression data resources available today. Increasing numbers of scholars have explored the GEO and have discovered large numbers of cancer-related lncRNAs, including MALAT1, AFAP1-AS1, and LINC00880 [8–10].

In this study, we screened the lncRNA A2M antisense RNA1 (A2M-AS1), a novel lncRNA that has not been previously reported, from GEO datasets and found that this lncRNA was obviously elevated in human breast cancer tissues. A2M-AS1 upregulation was significantly associated with ER-negative, HER2-positive, and basal-like breast cancer and with poor prognosis. Importantly, these results were validated in human breast cancer tissues that we collected. Functional experiments suggested that A2M-AS1 could dramatically promote cell proliferation, invasion, and migration. Further study showed that A2M-AS1 regulated signaling downstream of the cell adhesion molecule pathway by positively affecting CD2 and SELL expression. Our studies provide promising insight into A2M-AS1, a potential prognostic biomarker and therapeutic target for breast cancer.

## 2. Materials and Methods

### 2.1. GEO Datasets

The datasets were downloaded from the GEO. The datasets GSE45827 and GSE65216 were provided by the Institut Curie in France. The transcriptome analyses of the human breast cancer samples and adjacent normal breast tissue samples in these datasets were performed with Affymetrix Human Genome U133 Plus 2.0 Arrays [11]. GSE102484 was provided by the Koo Foundation SYS Cancer Centre in Taiwan. Expression profiling for this dataset was also conducted with an Affymetrix Human Genome U133 Plus 2.0 Array. According to the official description of GSE45827, the dataset contains 130 primary invasive breast cancer samples, including 126 invasive ductal carcinomas and 4 medullary carcinomas (41 triple-negative (TN), 30 HER2+, 29 Luminal A, and 30 Luminal B samples), as well as 11 normal tissue samples and 14 cell lines. GSE65216 (comprising GSE65194 and GSE65212) contains 306 primary invasive breast cancer samples, including 302 invasive ductal carcinomas and 4 medullary carcinomas (110 TN, 78 HER2+, 58 Luminal A, and 60 Luminal B samples), as well as 22 normal tissue samples and 14 cell lines. Only GSE65194 has related clinical data. GSE102484 contains only 683 primary breast cancer tissue samples.

### 2.2. Human Tissues and Cell Lines

Samples from breast cancer patients (tumour tissues and adjacent normal tissues) were obtained from the Affiliated Hospital of Jiangnan University; these samples were composed of 96 human primary breast cancer tissues and 64 paired adjacent noncancerous tissues. This project was authorized by the Research Ethics Committees of the corresponding institutions, and informed consent was obtained from all patients. In total, four human breast cancer cell lines were purchased from the American Type Culture Collection (ATCC; Manassas, VA, USA). The cell lines were characterized using short tandem repeat (STR) markers by the Genetic Testing Biotechnology Corporation (Suzhou, China). MDA-MB-231, ZR-75-30, and Hs578T cells were cultured in RPMI DMEM. BT-549 cells were cultured in RPMI 1640. These two complete media both included 10% foetal bovine serum and 1% streptomycin and penicillin. All materials were obtained from the same company (HyClone, Utah, USA). All cells were cultured at 37°C with 5% (v/v) CO_2_ in a humidified chamber.

### 2.3. RNA Isolation and Real-Time Quantitative PCR (qRT-PCR)

Total RNA was extracted from the breast cancer cell lines and breast cancer tissues using TRIzol reagent [12]. cDNA was generated with relevant primers (Supplementary Table 1). qRT-PCR was then performed using an Applied Biosystems system. All procedures were performed according to the manufacturer's protocols. The 2^−ΔΔCt^ method was used to analyse the relative gene expression data.

### 2.4. Transfection of Cell Lines

For small interfering RNA (siRNA) transfection, we designed three human A2M-AS1-specific targeting sequences, including GGATCCAGGTTGGTAGCAA (siRNA-A2M-AS1-1), CCTTTAATCCTGGCTCAAA (siRNA-A2M-AS1-2), and GGTGTAACATAGGACACAT (siRNA-A2M-AS1-3), with the A2M-AS1 sequence (Ensembl: ENSG00000245105) as a reference. A nontargeting negative control sequence was provided by RiboBio (Guangzhou, China). To create an A2M-AS1 expression vector, a pcDNA-GFP-Puro empty vector was purchased from Hanbio Biotechnology (Shanghai, China), and A2M-AS1 was cloned into the EcoRI and BamHI sites. According to the manufacturer's protocol, the siRNA and plasmid were transfected into breast cancer cell lines using Lipofectamine 2000 (Invitrogen, Carlsbad, CA, USA). After transfection for 24–72 h, the cells were used for the subsequent experiments.

### 2.5. Cell Counting Kit-8 (CCK-8) Assay

Briefly, breast cancer cells were seeded in 96-well plates (800 cells per plate). According to the manufacturer's instructions, 10 *μ*l of CCK-8 reagent (Dojindo Molecular Technologies, Kumamoto, Japan) mixed with 90 *μ*l of serum-free medium was added to each well. The absorbance was measured at 450 nm.

### 2.6. Transwell Matrigel Invasion Assay and Migration Assay

The breast cancer cell lines were cultured in 24-well plates. The membrane pore diameter was 8 mm. For the invasion assay, 60 *μ*l of Matrigel was added to the upper chamber, 800 *μ*l of medium with 20% foetal bovine serum was added to the lower chamber, and the plates were cultured at 37°C with 5% (v/v) CO_2_ in a humidified chamber for 2 h. Subsequently, cells (2 × 10^4^ cells per plate) were seeded into the upper chamber with 300 *μ*l of serum-free medium. For the migration assay, cells (2 × 10^4^ cells per plate) in 300 *μ*l of serum-free medium were seeded into the upper chamber without Matrigel, and 800 *μ*l of medium with 20% foetal bovine serum was added to the lower chamber. For both the invasion and migration assays, after culturing for 12–24 h at 37°C in a humidified chamber with 5% (v/v) CO_2_, the upper chambers were washed with PBS 3 times, and the cells were stained with crystal violet. Then, the cells on the upper part of the membrane were wiped away, and images of 3 random fields were captured under a microscope.

### 2.7. Scratch Wound Healing Assay

The breast cancer cell lines were cultured in 6-well plates with 0-1% FBS until they reached 95–100% confluence. Using a 200-*μ*l pipette tip, 3 scratches were made. After 24–72 h, 3 random fields were photographed when the experimental group healing rates reached at least 70%, as determined by microscopy.

### 2.8. Western Blotting

Protein was extracted from cells with lysis buffer, and the concentration was measured with a bicinchoninic acid (BCA) protein assay. The total protein was subjected to 10% SDS-PAGE (Beyotime) for approximately 90 min, and the separated proteins were transferred to immobilon membranes (PVDF, Millipore) for approximately 60 min. The membranes were incubated with 10% nonfat milk for 1 h at room temperature and then incubated with primary antibodies against CD2 (1 : 1000, goat anti-rabbit antibody, Abcam, Shanghai, China), SELL (1 : 1000, goat anti-rabbit antibody, Abcam, Shanghai, China), and GAPDH (1 : 1000, mouse anti-rabbit antibody, CST, MA, USA) separately at 4°C overnight. Then, the membranes were incubated with secondary antibodies (1 : 800, rabbit anti-mouse antibody, CST, MA, USA) for 2 h. Finally, the proteins were detected using an ECL Western blot kit (EnoGene, Shanghai, China). Images were captured using Sigmatel software v2.0, and Image Lab software v3.0 was used to calculate the density of the protein bands.

### 2.9. Statistical Analysis

Statistical Package for the Social Sciences 21.0 (SPSS 21.0; International Business Machines Corporation, NY, USA) was used to conduct the statistical analyses. Differentially expressed genes (DEGs) were analysed using the limma package in R version 3.5.1. Heatmaps [13] were generated with the pheatmap package in R version 3.5.1. Differences with *P* values ≤0.001 and |log 2-fold change (FC)| values >2 were regarded as statistically significant. All experiments were performed independently and in triplicate, and two-tailed Student's *t*-tests were applied to assess the statistical significance of differences between two independent groups. The data are presented as the mean ± standard deviation.

## 3. Results

### 3.1. Differentially Expressed lncRNAs in Human Breast Cancer

**Figure 1:**
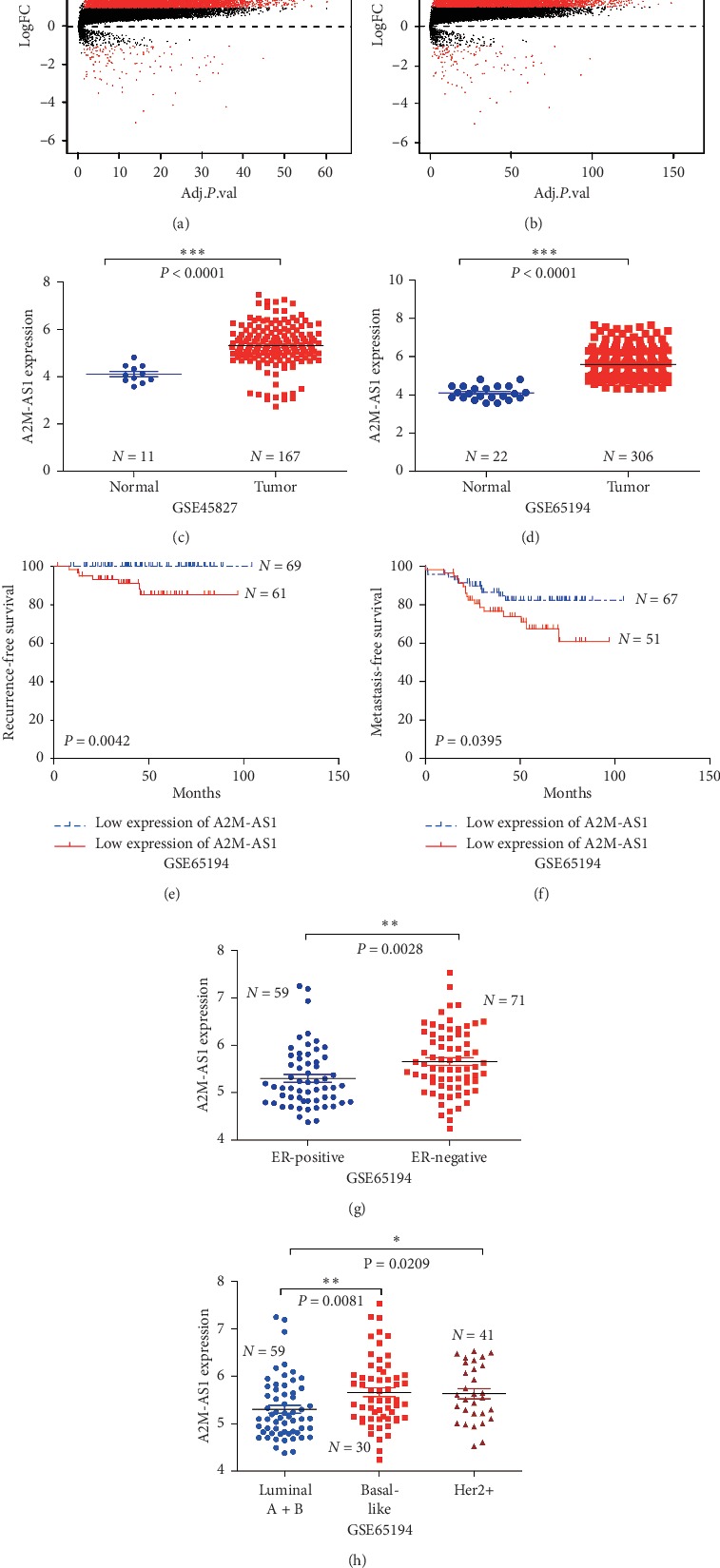
A2M-AS1 is upregulated in breast cancer tissues in the GEO dataset and associated with worse prognosis. (a) Volcano plot of 399 dysregulated lncRNAs and 11070 dysregulated mRNAs identified by hierarchical clustering in GSE45827. (b) Volcano plot of 383 dysregulated lncRNAs and 11164 dysregulated mRNAs identified by hierarchical clustering in GSE65216. A2M-AS1 expression in human breast cancer tumour tissues and normal tissues was detected by RNA microarray using GSE45827 (c) and GSE65216 (d). High expression of A2M-AS1 is associated with poor recurrence-free (e) and metastasis-free survival (f) rates. (g) A2M-AS1 is more highly expressed in ER-negative tissues than in ER-positive tissues. (h) A2M-AS1 is more highly expressed in basal-like and Her2+ tissues than in luminal A and luminal B tissues. Notes: ^*∗*^*P* < 0.05, ^*∗∗*^*P* < 0.01, and ^*∗∗∗*^*P* < 0.001.

To identify differentially expressed lncRNAs in human breast cancer tissues, we measured RNA expression with an Affymetrix Human Genome U133 Plus 2.0 RNA microarray. In the expression profile for dataset GSE45827, 153 primary invasive breast cancer and 11 normal tissue samples were investigated, and 399 lncRNAs were found to be differentially expressed between the tumour group and the normal group (*P* < 0.001) (Figure 1(a)). In total, 7 downregulated lncRNAs and 392 upregulated lncRNAs (with ≥2.0-fold changes) were identified. Furthermore, analysis of the GSE65216 expression data, including 306 breast cancer and 22 nontumour samples, revealed 383 differentially expressed lncRNAs (Figure 1(b)). Seven downregulated lncRNAs and 376 upregulated lncRNAs (with ≥2.0-fold changes) were identified. The two datasets had 368 differentially expressed lncRNAs in common, including 7 downregulated lncRNAs and 361 upregulated lncRNAs. The data for the differentially expressed mRNAs are not shown.

### 3.2. A2M-AS1 Promotes Tumour Recurrence and Metastasis in Breast Cancer

**Figure 2:**
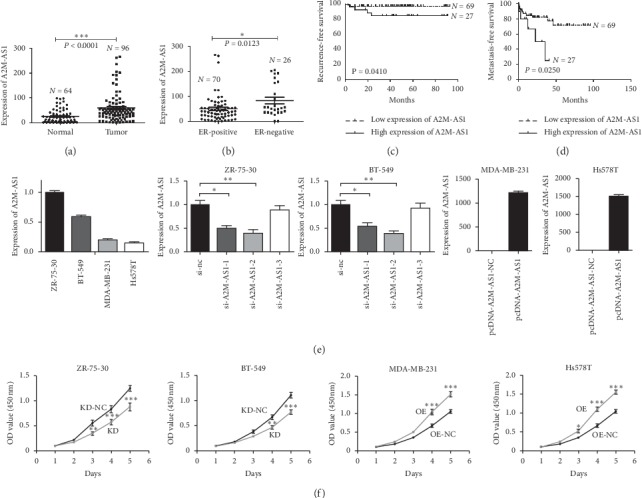
A2M-AS1 is upregulated in breast cancer tissues and promotes breast cancer cell proliferation. (a) A2M-AS1 expression in breast cancer tissues and normal tissues was measured by qRT-PCR. (b) A2M-AS1 expression was detected in ER-negative and ER-positive breast cancer tissues. Kaplan–Meier analysis of recurrence-free (c) and metastasis-free (d) survival in two groups divided according to low and high expression levels of A2M-AS1. (e) qRT-PCR revealed endogenous expression of A2M-AS1 and the efficiency of A2M-AS1 knockdown and overexpression in four ER-negative breast cancer cell lines. (f) The effect of A2M-AS1 on cell proliferation was detected by CCK8 assays in ZR-75-30/BT-549 cells with A2M-AS1 knockdown and in MDA-MB-231/Hs578T cells with A2M-AS1 overexpression. GAPDH was used as the internal reference. Data are presented as the mean ± SD of three independent experiments. Notes: KD: A2M-AS1-knockdown group; KD-NC: negative control for the A2M-AS1-knockdown group; OE: A2M-AS1-overexpression group; OE-NC: negative control for the A2M-AS1-overexpression group. ^*∗*^*P* < 0.05, ^*∗∗*^*P* < 0.01, and ^*∗∗∗*^*P* < 0.001.

In our screening experiments, we identified a novel lncRNA, A2M-AS1, that has not been previously reported. A2M-AS1 was upregulated in breast cancer tissues compared with their paired adjacent noncancerous tissues (Figures 1(c) and 1(d)). Through combined analysis of expression data and clinical data, we found that higher expression levels of A2M-AS1 were associated with worse recurrence-free and metastasis-free survival in patients with breast cancer (Figures 1(e) and 1(f)). Further research on the relationship between A2M-AS1 and clinicopathological parameters revealed that, compared with ER-positive tissues, ER-negative tissues overexpressed A2M-AS1 (*P* < 0.0028) (Figure 1(g)). A2M-AS1 was also more highly expressed in basal-like tissues (*P* < 0.0081) and HER2+ tissues (*P* < 0.0209) than in luminal A/luminal B tissues (Figure 1(h)). There was no significant difference in A2M-AS1 expression between basal-like and HER2+ tissues (*P* > 0.05). We collected 96 human breast cancer primary tumour tissues and 64 paired adjacent noncancerous tissues from the Affiliated Hospital of Jiangnan University to validate the results of the GEO analysis. As anticipated, A2M-AS1 was significantly upregulated in the breast cancer tumour tissues compared with the paired adjacent noncancerous tissues (Figure 2(a)). In addition, compared with ER-positive tissues, ER-negative tissues overexpressed A2M-AS1 (Figure 2(b)). Analysis of the follow-up data revealed that higher expression levels of A2M-AS1 were significantly associated with shorter recurrence-free survival and metastasis-free survival in patients with breast cancer (*P* < 0.05) (Figures 2(c) and 2(d)). Collectively, these data demonstrate that A2M-AS1 is upregulated in breast cancer tissue, especially in ER-negative tissues, and promotes breast tumour recurrence and metastasis.

### 3.3. A2M-AS1 Promotes Breast Cancer Cell Proliferation, Invasion, and Migration In Vitro

qRT-PCR was used to analyse the endogenous expression of A2M-AS1 in human breast cancer cell lines (BT-549, ZR-75-30, Hs578T, and MDA-MB-231). Among these cell lines, BT-549 and ZR-75-30 had higher levels of A2M-AS1 expression than Hs578T and MDA-MB-231. We knocked down A2M-AS1 in ZR-75-30 and BT-549 cells by separately transfecting the cells with three siRNAs (si-A2M-AS1-1, si-A2M-AS1-2, and si-A2M-AS1-3), and we overexpressed A2M-AS1 in MDA-MB-231 and Hs578T cells by transfecting the cells with pcDNA-A2M-AS1. The efficiency of knockdown and overexpression was confirmed by qRT-PCR (Figure 2(e)).

**Figure 3:**
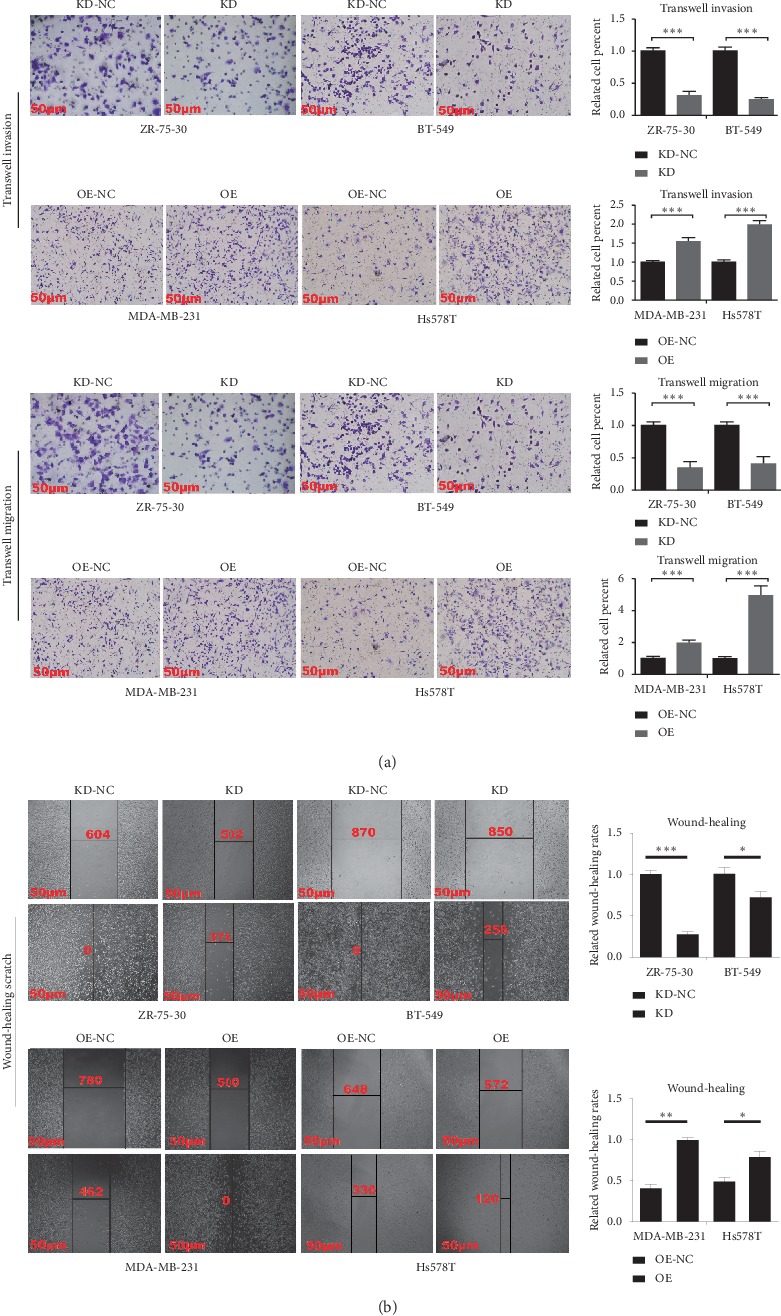
A2M-AS1 promotes breast cancer cell invasion and migration. (a) Cell invasion and migration were detected in ZR-75-30/BT-549 cells knocked down for A2M-AS1 and in MDA-MB-231/Hs578T cells overexpressing A2M-AS1 via Transwell Matrigel invasion and migration assays. (b) Scratch wound-healing assays were used to measure cell migration in ZR-75-30/BT-549 cells knocked down for A2M-AS1 and in MDA-MB-231/Hs578T cells overexpressing A2M-AS1. Data are presented as the mean ± SD of three independent experiments. Notes: KD: A2M-AS1-knockdown group; KD-NC: negative control for the A2M-AS1-knockdown group; OE: A2M-AS1-overexpression group; OE-NC: negative control for the A2M-AS1-overexpression group. ^*∗*^*P* < 0.05, ^*∗∗*^*P* < 0.01, and ^*∗∗∗*^*P* < 0.001.

The CCK-8 assay revealed that downregulation of A2M-AS1 with si-A2M-AS1-2 reduced ZR-75-30 and BT-549 cell proliferation, while upregulation of A2M-AS1 enhanced MDA-MB-231 and Hs578T cell proliferation (Figure 2(f)). We also knocked down A2M-AS1 and performed transwell invasion and migration assays to study the invasion and migration abilities of ZR-75-30 and BT-549 cells. The groups of ZR-75-30 and BT-549 cells with A2M-AS1 knockdown exhibited less invasion and migration than the negative control groups, while the groups of MDA-MB-231 and Hs578T cells overexpressing A2M-AS1 exhibited greater invasion and migration than the negative control groups (Figure 3(a)). Additionally, scratch wound healing assays were performed and revealed that downregulation of A2M-AS1 reduces ZR-75-30 and BT-549 cell migration, while upregulation of A2M-AS1 enhances MDA-MB-231 and Hs578T cell migration (Figure 3(b)). In summary, A2M-AS1 significantly promotes breast cancer cell invasion and migration.

### 3.4. Identification of the Relationships between A2M-AS1 and Coexpressed Genes in Breast Cancer

**Figure 4:**
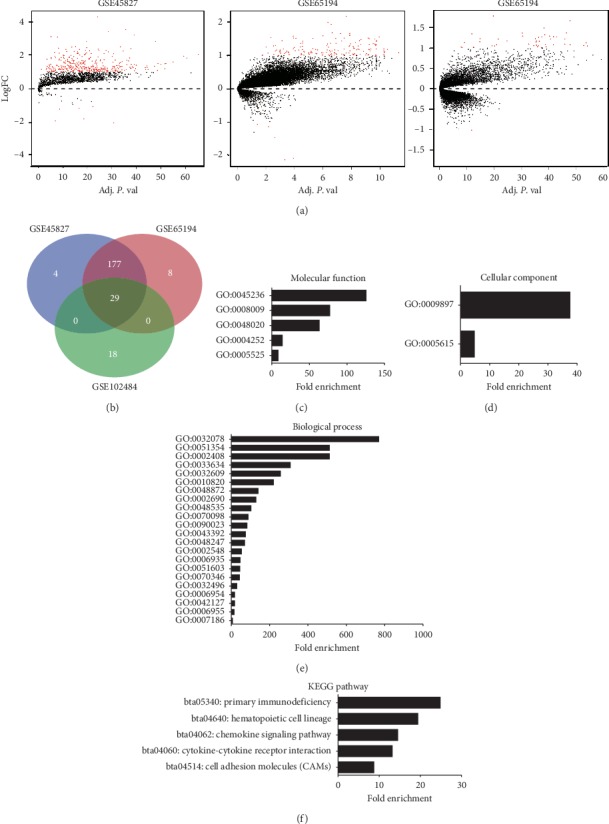
Coexpression relationships between A2M-AS1 and genes in breast cancer in the GEO database and GO function and KEGG pathway analyses. (a) Volcano plot of 211 genes coexpressed with A2M-AS1 in GSE45827, 215 genes coexpressed with A2M-AS1 in GSE65194, and 48 genes coexpressed with A2M-AS1 in GSE102484. (b) The 29 genes coexpressed with A2M-AS1 and common to all three GEO datasets are shown in the Venn diagram. (c–e) GO functional analysis of the 29 genes coexpressed with A2M-AS1 by David. (f) KEGG pathway analysis of the genes coexpressed with A2M-AS1 by David. *P* < 0.01, log FC ≥1.5 was regarded as significant coexpression.

To elucidate the potential molecular mechanisms of A2M-AS1 in breast cancer, we investigated genes that may be coexpressed with A2M-AS1. We divided the GSE45827 and GSE65194 samples into two groups according to the average expression levels of A2M-AS1. Among the GSE45827 mRNA expression data, a total of 211 DEGs were identified in the high A2M-AS1 expression group (*N* = 89) compared with the low A2M-AS1 expression group (*N* = 89) (Figure 4(a)). Among the GSE65194 mRNA expression data, 215 DEGs were identified in the high A2M-AS1 expression group (*N* = 89) compared with the low A2M-AS1 expression group (*N* = 89) (Figure 4(a)). To exclude differences between normal tissues and tumour tissues, we analysed GSE102484 mRNA expression data (containing only tumour sample data). We grouped these samples into two groups according to the mean expression level of A2M-AS1. A total of 47 DEGs were found between the groups with low (*N* = 360) and high (*N* = 323) A2M-AS1 expression levels (Figure 4(a)). Among the three GEO datasets, 29 DEGs were shared (Figure 4(b)). These 29 DEGs were determined to potentially be regulated by A2M-AS1 (Supplement Table 2); however, the exact mechanisms and interactions among them remained unclear. Therefore, we performed additional studies.

### 3.5. Bioinformatics Analysis of the Genes Coexpressed with A2M-AS1 in Breast Cancer

For bioinformatics analysis, Gene Ontology (GO) functional analysis and Kyoto Encyclopedia of Genes and Genomes (KEGG) pathway analysis were performed on the Database for Annotation, Visualization and Integrated Discovery (David) website (https://david.ncifcrf.gov/). Among GO terms in the molecular function category, the DEGs were significantly enriched for the chemokine activity, GTP binding, CXCR chemokine receptor binding, serine-type endopeptidase activity, and CCR chemokine receptor binding terms (Figure 4(c)). In the cellular component category, the DEGs were significantly enriched for the external side of plasma membrane and extracellular space terms (Figure 4(d)). In addition, the most enriched GO terms in the biological process category were the positive regulation of cell-cell adhesion mediated by integrin, myeloid dendritic cell chemotaxis, negative regulation of endodeoxyribonuclease activity, proteolysis involved in cellular protein catabolic process, and regulation of cell proliferation terms, among others (Figure 4(e)). KEGG pathway analysis revealed that the DEGs were enriched in the cell adhesion molecules, cytokine-cytokine receptor interaction, primary immunodeficiency, and chemokine signaling pathways (Figure 4(f)).

### 3.6. A2M-AS1 Promotes the Invasion and Migration of Breast Cancer Cells by Regulating Downstream Factors in the Cell Adhesion Molecule Pathway

In our studies, we found that DEGs coexpressed with A2M-AS1, including CD2, CD8A and SELL, were significantly enriched in the cell adhesion molecule pathway (Figure 4(f)). Analysis of the GSE45827, GSE65194, and GSE102484 mRNA expression data revealed that A2M-AS1 expression was significantly positively correlated with CD2, CD8A, and SELL expression (Supplement Figures 1(a)–1(c)). Further KEGG analysis revealed that CD2 and SELL were in the same pathway (the leukocyte transendothelial migration pathway) (Supplementary Figure 2). Therefore, we chose these two molecules for verification.

**Figure 5:**
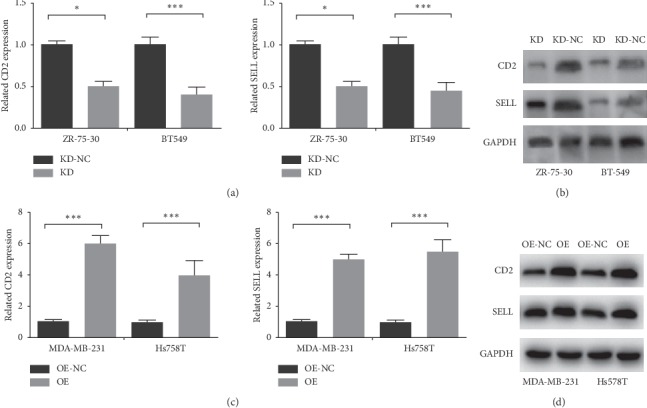
CD2 and SELL are positively correlated with A2M-AS1 in breast cancer cell lines. After knockdown in ZR-75-30/BT-59 cells lines, CD2 and SELL mRNA and protein levels were determined by qRT-PCR (a) and western blotting (b). After overexpression of A2M-AS1 in MDA-MB-231/Hs578T cells lines, CD2 and SELL mRNA and protein levels were determined by qRT-PCR (c) and western blotting (d). Data are presented as the mean ± SD of three independent experiments. GAPDH was used as the internal reference. Notes: KD: A2M-AS1-knockdown group; KD-NC: negative control for the A2M-AS1-knockdown group; OE: A2M-AS1-overexpression group; OE-NC: negative control for the A2M-AS1-overexpression group. ^*∗*^*P* < 0.05, ^*∗∗*^*P* < 0.01, and ^*∗∗∗*^*P* < 0.001.

We knocked down A2M-AS1 in ZR-75-30 and BT-549 cells and performed qRT-PCR and Western blot analyses. We found that knockdown of A2M-AS1 reduced the expression of CD2 and SELL in these cells (Figures 5(a) and 5(b)); similarly, overexpression of A2M-AS1 increased the expression of CD2 and SELL in MDA-MB-231 and Hs578T cells (Figures 5(c) and 5(d)). In summary, A2M-AS1, by regulating the expression of CD2 and SELL, promotes invasion and migration in breast cancer.

## 4. Discussion

Despite significant advances in the treatment of breast cancer over the past decade, patient outcomes are still worth considering due to the high incidence of cancer-specific deaths; in addition, the molecular mechanisms of breast cancer remain unclear [14]. Accumulating documents have suggested that lncRNAs play crucial roles during tumorigenesis and cancer progression [15]. The mechanisms by which lncRNAs participate in breast cancer may represent key nodes for therapeutic intervention [4].

In this study, by integrating the expression profiles of the GEO datasets GSE45827 and GSE65216, we identified hundreds of differentially expressed lncRNAs, including HOTAIR, ASAP1-IT1, TUG1, and MIAT, which have been reported to be related to cancer by other investigators [16–19]. Of these differentially expressed lncRNAs, A2M-AS1, a novel lncRNA, was upregulated in breast cancer tissues and was associated with poor prognosis. A previous study reported that A2M upregulation is related to metastasis in osteosarcoma and to ovarian carcinogenesis [20, 21]. A2M-AS1, the antisense of A2M, is located on the forward strand of chromosome 12 at positions 9,065,177–9,068,684 and may be similar to A2M. We validated A2M-AS1 expression in 96 breast cancer tissues and 64 paired normal tissues collected from the Affiliated Hospital of Jiangnan University, confirming that A2M-AS1 is upregulated in breast cancer. In clinically relevant analyses of both GEO samples and our collected samples, we found that A2M-AS1 was significantly associated with ER-negative breast cancer and that increased expression of A2M-AS1 was associated with reduced recurrence-free survival and metastasis-free survival rates in patients with breast cancer.

Identification of ER status is useful for clinical hormonotherapy and prognostic prediction in breast cancer. Early reports showed that ER-negative breast cancer is generally more aggressive than ER-positive breast cancer [22, 23]. Recently, reports have shown that the basal-like subtype, characterized by negativity for ER, is associated with aggressive histology, poor prognosis, and resistance to endocrine therapies [24–26]. Therefore, we selected 4 ER-negative and basal-like breast cancer cell lines and performed further experiments.

Functional experiments showed that A2M-AS1 enhances breast cancer cell proliferation, invasion, and migration, and bioinformatics analysis showed that A2M-AS1 potentially regulates cell adhesion molecule pathway members, including CD2, CD8A, and SELL. Cell adhesion molecules are members of the immunoglobulin superfamily. Previous researchers have reported that abnormal expression of cell adhesion molecules plays a major role in the progression of various human cancers. For example, in oral carcinoma, upregulation of MGAT5 plays an active role in disease progression [27]. In addition, CADM2 inhibits tumour progression in prostate cancer [28]. The leukocyte transendothelial migration pathway is associated with the cell adhesion molecule pathway, and dysregulation of this pathway is related to various types of disease, including gastric cancer, papillary thyroid carcinoma, and acute myocardial infarction [29, 30]. CD2 and SELL participate in the leukocyte transendothelial migration pathway. CD2, which is regarded as an immune response molecule, has been documented to be involved in breast cancer recurrence and metastasis and to play a vital role as a prognostic factor for patients with breast cancer [31–33]. SELL is a cell surface adhesion molecule. Immunodepletion of SELL decreases the migration of MDA-MB-231 cells [34]. IL-33 inhibits adhesion and invasion via downregulation of SELL in trophoblast cell lines [35]. We found that knockdown of A2M-AS1 decreased the mRNA and protein expression levels of CD2 and SELL. In contrast, overexpression of A2M-AS1 increased the mRNA and protein expression levels of CD2 and SELL. These results reveal that A2M-AS1 regulates the expression of CD2 and SELL, thereby promoting breast cancer cell invasion and migration.

In summary, our present study shows that A2M-AS1 is upregulated in breast cancer and is associated with poor prognosis. A2M-AS1 increases breast cancer cell invasion and migration by regulating the cell adhesion molecules CD2 and SELL; therefore, our findings provide a potential prognostic biomarker and therapeutic target for breast cancer.

## Data Availability

The data sets used and analysed in the current study are available upon appropriate request from the authors.

## Supplementary Materials

Supplementary Materials Supplement Table 1: the primers that were used in this study. Supplement Table 2: the 29 genes coexpressed with A2M-AS1 that were common among the GSE45827, GSE65194, and GSE102484 datasets. Supplement Table 3: details of 29 genes coexpression with A2M-AS1 in GO and KEGG pathways. Supplement Figure 1: downstream of the cell adhesion molecule pathway, CD2, CD8A, and SELL were coexpressed with A2M-AS1 according to GEO datasets. (a–c) Pearson's correlation coefficient was used to evaluate correlations between A2M-AS1 and CD2, CD8A, and SELL in GSE45827, GSE65194, and GSE102484. ^*∗*^*P* < 0.05, ^*∗∗*^*P* < 0.01, ^*∗∗∗*^*P* < 0.001. Supplement Figure 2: downstream of the cell adhesion molecule pathway and genes coexpressed with A2M-AS1 that are involved in the cell adhesion molecule pathway. Notes: red represents that the gene is positively controlled by A2M-AS1; green represents that the gene is negatively controlled by A2M-AS1.
