# Supplementary material for: Screening of a Novel Upregulated lncRNA, A2M-AS1, That Promotes Invasion and Migration and Signifies Poor Prognosis in Breast Cancer
Source: Biomed Res Int. 2020 Apr 11;2020:9747826. doi: 10.1155/2020/9747826 (PMC7171613; doi:10.1155/2020/9747826)
Supplement: Supplementary Materials — Supplement Table 1: the primers that were used in this study. Supplement Table 2: the 29 genes coexpressed with A2M-AS1 that were common among the GSE45827, GSE65194, and GSE102484 datasets. Supplement Table 3: details of 29 genes coexpression with A2M-AS1 in GO and KEGG pathways. Supplement Figure 1: downstream of the cell adhesion molecule pathway, CD2, CD8A, and SELL were coexpressed with A2M-AS1 according to GEO datasets. (a–c) Pearson's correlation coefficient was used to evaluate correlations between A2M-AS1 and CD2, CD8A, and SELL in GSE45827, GSE65194, and GSE102484. ∗P < 0.05, ∗∗P < 0.01, ∗∗∗P < 0.001. Supplement Figure 2: downstream of the cell adhesion molecule pathway and genes coexpressed with A2M-AS1 that are involved in the cell adhesion molecule pathway. Notes: red represents that the gene is positively controlled by A2M-AS1; green represents that the gene is negatively controlled by A2M-AS1. [file 9747826.f1.zip › 9747826.f1/Supplement Table 2.doc]

Supplement Table 2: The 29 genes co-expression with A2M-AS1 that were common among the GSE45827, GSE65194 and GSE102484 datasets
Gene	GSE45827		GSE65194		GSE102484		
	logFC	P.Value	logFC	P.Value	logFC	P.Value	
C16orf54	1.408466976	1.15E-14	1.402653091	9.59E-15	1.283357283	1.37E-43	
CCL19	1.738513103	5.59E-13	1.249126982	6.00E-13	1.656288263	1.07E-51	
CCL5	1.344196344	3.17E-09	1.359355869	2.05E-09	1.140954013	2.65E-38	
CCR7	1.360773175	5.33E-11	1.011208773	7.97E-11	1.262365681	1.54E-40	
CD2	1.533704437	1.77E-12	1.544232963	1.40E-12	1.042752979	2.24E-37	
CD52	1.808130989	7.18E-15	1.095088087	4.65E-15	1.268075917	3.60E-45	
CD69	1.224187345	2.76E-09	1.477555014	1.77E-09	1.0831145	1.77E-35	
CD8A	1.161020845	5.67E-10	1.169722136	5.02E-10	1.026993667	7.56E-39	
COL11A1	1.474496099	1.40E-05	1.287225602	1.31E-05	1.031151315	1.11E-13	
CXCL13	1.884072608	2.93E-07	1.064887357	1.58E-07	1.129623124	6.22E-11	
CXCL9	2.460717133	3.53E-12	1.169173342	6.61E-12	1.141960499	7.50E-17	
ENPP2	1.047060867	1.65E-07	1.076364108	1.07E-07	1.078680315	6.68E-42	
EOMES	1.09526689	7.48E-12	1.20328689	1.96E-11	1.083118016	1.25E-49	
FDCSP	1.728889316	0.00011954	1.395055765	4.06E-05	1.771187334	5.61E-22	
GIMAP7	1.25078215	2.90E-11	1.244335431	4.26E-11	1.118898534	5.87E-58	
GPR171	1.440599961	9.66E-15	1.893255932	8.97E-15	1.251910552	2.81E-39	
GZMA	1.536459449	3.50E-13	1.537013634	3.81E-13	1.165411062	1.83E-38	
GZMK	1.665798733	3.60E-15	1.615188388	3.72E-15	1.363988263	2.58E-50	
IGHM	1.6659409	2.94E-13	1.038960277	3.30E-13	1.04205114	1.53E-22	
IGJ	2.067733541	5.39E-08	1.213507608	2.90E-08	1.209489071	7.05E-24	
IGLL5	1.925776299	4.88E-14	1.416922491	4.59E-14	1.043568114	1.62E-21	
IL7R	1.437536314	1.35E-13	1.286830993	1.45E-13	1.214376954	1.40E-49	
ITK	1.460173265	9.03E-15	1.460996233	8.03E-15	1.315972581	1.10E-41	
LAMP3	1.286629318	1.77E-06	1.201941908	8.61E-07	1.076020517	3.10E-23	
MS4A1	1.114116744	1.97E-11	2.326360807	3.81E-11	1.478069856	2.41E-41	
SELL	1.539231405	7.23E-13	1.639574078	8.28E-13	1.269114283	3.28E-45	
TNFRSF17	1.232617459	1.80E-09	1.233390388	1.62E-09	1.165582267	3.17E-27	
TRBC1	1.798738841	6.31E-14	1.456709481	5.67E-14	1.262761622	2.40E-46	
ZC3H12D	1.106450076	3.59E-13	1.307632931	4.63E-13	1.002205366	2.61E-40	
