# Supplementary material for: Screening of a Novel Upregulated lncRNA, A2M-AS1, That Promotes Invasion and Migration and Signifies Poor Prognosis in Breast Cancer
Source: Biomed Res Int. 2020 Apr 11;2020:9747826. doi: 10.1155/2020/9747826 (PMC7171613; doi:10.1155/2020/9747826)
Supplement: Supplementary Materials — Supplement Table 1: the primers that were used in this study. Supplement Table 2: the 29 genes coexpressed with A2M-AS1 that were common among the GSE45827, GSE65194, and GSE102484 datasets. Supplement Table 3: details of 29 genes coexpression with A2M-AS1 in GO and KEGG pathways. Supplement Figure 1: downstream of the cell adhesion molecule pathway, CD2, CD8A, and SELL were coexpressed with A2M-AS1 according to GEO datasets. (a–c) Pearson's correlation coefficient was used to evaluate correlations between A2M-AS1 and CD2, CD8A, and SELL in GSE45827, GSE65194, and GSE102484. ∗P < 0.05, ∗∗P < 0.01, ∗∗∗P < 0.001. Supplement Figure 2: downstream of the cell adhesion molecule pathway and genes coexpressed with A2M-AS1 that are involved in the cell adhesion molecule pathway. Notes: red represents that the gene is positively controlled by A2M-AS1; green represents that the gene is negatively controlled by A2M-AS1. [file 9747826.f1.zip › 9747826.f1/Supplement Table 1.doc]

**Supplement Table 1: The primers that were used in this study**

| **Gene names** | **Primer** |
| --- | --- |
| GAPDH | Forward 5’-CAATGACCCCTTCATTGACC-3’  Reverse 5’-GACAAGCTTCCCGTTCTCAG-3’ |
| A2M-AS1 | Forward 5’-TGTCGCAATACTTACTGCAAAGA-3’  Reverse 5’-AGGATTCATGCAGAAGCACG-3’ |
| CD2 | Forward 5’-GCCAGAGTAATGGGCTCTCTG-3’  Reverse 5’-CGGCCCCTTTCTTCAGTAGC-3’ |
| SELL | Forward 5’-CCACCTGTGGACCATTTGGA-3’  Reverse 5’-CCAGGGGATGGCTACAGTTC-3’ |
