# Supplementary material for: Screening of a Novel Upregulated lncRNA, A2M-AS1, That Promotes Invasion and Migration and Signifies Poor Prognosis in Breast Cancer
Source: Biomed Res Int. 2020 Apr 11;2020:9747826. doi: 10.1155/2020/9747826 (PMC7171613; doi:10.1155/2020/9747826)
Supplement: Supplementary Materials — Supplement Table 1: the primers that were used in this study. Supplement Table 2: the 29 genes coexpressed with A2M-AS1 that were common among the GSE45827, GSE65194, and GSE102484 datasets. Supplement Table 3: details of 29 genes coexpression with A2M-AS1 in GO and KEGG pathways. Supplement Figure 1: downstream of the cell adhesion molecule pathway, CD2, CD8A, and SELL were coexpressed with A2M-AS1 according to GEO datasets. (a–c) Pearson's correlation coefficient was used to evaluate correlations between A2M-AS1 and CD2, CD8A, and SELL in GSE45827, GSE65194, and GSE102484. ∗P < 0.05, ∗∗P < 0.01, ∗∗∗P < 0.001. Supplement Figure 2: downstream of the cell adhesion molecule pathway and genes coexpressed with A2M-AS1 that are involved in the cell adhesion molecule pathway. Notes: red represents that the gene is positively controlled by A2M-AS1; green represents that the gene is negatively controlled by A2M-AS1. [file 9747826.f1.zip › 9747826.f1/Supplement Table 3.doc]

Supplement Table 3: Details of 29 genes co-expression with A2M-AS1 in GO and KEGG pathways

| GO and KEGG | Genes |
| --- | --- |
| GO:0070098~chemokine-mediated signaling pathway | CCR7, CXCL13, CXCL9, CCL19, CCL5 |
| GO:0006954~inflammatory response | CCR7, CXCL13, CXCL9, CCL19, CCL5 |
| GO:0006935~chemotaxis | CCR7, ENPP2, CXCL9 |
| GO:0006955~immune response | CCR7, CXCL13, ENPP2, CXCL9 |
| GO:0032078~negative regulation of endodeoxyribonuclease activity | GZMA |
| GO:0002408~myeloid dendritic cell chemotaxis | CCR7, CCL19 |
| GO:0051354~negative regulation of oxidoreductase activity | GZMA |
| GO:0032496~response to lipopolysaccharide | CCR7, CXCL13, CXCL9 |
| GO:0033634~positive regulation of cell-cell adhesion mediated by integrin | CXCL13, CCL5 |
| GO:0032609~interferon-gamma production | ITK, EOMES |
| GO:0010820~positive regulation of T cell chemotaxis | CXCL13, CCL5 |
| GO:0007186~G-protein coupled receptor signaling pathway | CCR7, CXCL13, CXCL9, CCL19, CCL5 |
| GO:0048872~homeostasis of number of cells | CCR7, IL7R |
| GO:0042127~regulation of cell proliferation | ITK, CXCL13, CXCL9 |
| GO:0002690~positive regulation of leukocyte chemotaxis | CXCL13, CXCL9 |
| GO:0048535~lymph node development | CXCL13, IL7R |
| GO:0090023~positive regulation of neutrophil chemotaxis | CCR7, CCL19 |
| GO:0071356~cellular response to tumor necrosis factor | CCL19, CCL5 |
| GO:0071347~cellular response to interleukin-1 | CCL19, CCL5 |
| GO:0071346~cellular response to interferon-gamma | CCL19, CCL5 |
| GO:0051603~proteolysis involved in cellular protein catabolic process | GZMA |
| GO:0048247~lymphocyte chemotaxis | CCL19, CCL5 |
| GO:0043392~negative regulation of DNA binding | GZMA |
| GO:0030593~neutrophil chemotaxis | CCL19, CCL5 |
| GO:0007267~cell-cell signaling | CXCL13, CCL5 |
| GO:0002548~monocyte chemotaxis | CCL19, CCL5 |
| GO:0009897~external side of plasma membrane | CCR7, CD8A, CD69, SELL, CXCL9, CD2, MS4A1, IL7R |
| GO:0005623~cell | CXCL13, CCL19 |
| GO:0005615~extracellular space | CXCL13, ENPP2, CXCL9, MS4A1, CCL19, CCL5 |
| GO:0008009~chemokine activity | CXCL13, CXCL9, CCL19, CCL5 |
| GO:0005525~GTP binding | GIMAP7 |
| GO:0045236~CXCR chemokine receptor binding | CXCL13, CXCL9 |
| GO:0004252~serine-type endopeptidase activity | GZMK, GZMA |
| GO:0048020~CCR chemokine receptor binding | CCL19, CCL5 |
| KEGG:bta04060:Cytokine-cytokine receptor interaction | CCR7, CXCL13, CXCL9, TNFRSF17, CCL19, CCL5, IL7R |
| KEGG:bta04062:Chemokine signaling pathway | ITK, CCR7, CXCL13, CXCL9, CCL19, CCL5 |
| KEGG:bta04640:Hematopoietic cell lineage | CD8A, CD2, MS4A1, IL7R |
| KEGG:bta04514:Cell adhesion molecules (CAMs) | CD8A, SELL, CD2 |
| KEGG:bta05340:Primary immunodeficiency | CD8A, IL7R |
